# Evoked potential studies for predicting functional recovery in a case of acute necrotizing encephalopathy

**DOI:** 10.1002/ccr3.1473

**Published:** 2018-03-08

**Authors:** Yuya Onozawa, Toshiyuki Iwasaki, Takahiro Iizuka, Yutaka Nonoda, Taira Toki, Susumu Obata, Shinichi Munekata, Yuhsaku Kanoh

**Affiliations:** ^1^ Department of Clinical Laboratory Kitasato University Hospital Sagamihara Japan; ^2^ Department of Pediatrics Kitasato University School of Medicine Sagamihara Japan; ^3^ Department of Neurology Kitasato University School of Medicine Sagamihara Japan; ^4^ Department of Laboratory Medicine Kitasato University School of Medicine Sagamihara Japan

**Keywords:** Acute necrotizing encephalopathy, electroencephalogram, evoked potentials, MRI

## Abstract

Normal‐appearing evoked potentials during the acute stage of the disease despite persistent coma may predict subsequent functional recovery of the brain in a pediatric case of acute necrotizing encephalopathy, indicating that evoked potential studies are useful for predicting functional outcome of the brain.

## Introduction

We report a 7‐year‐old boy admitted with status epilepticus due to acute necrotizing encephalopathy (ANE). Evoked potentials appeared normal despite persistent coma. After intensive treatment, he had significantly recovered. Normal‐appearing evoked potentials despite persistent coma may predict subsequent functional recovery of the brain in a pediatric case of ANE.

Acute necrotizing encephalopathy is a severe form of acute encephalopathy characterized by diffuse brain edema and bilateral thalamic lesions 1. This condition predominantly affects children in Asian and Western countries, with an estimated mortality rate of 30% 2, 3. This disorder frequently causes significant disability with intellectual deficits and epileptic seizures as residual neurological deficits. The presence of burst‐suppression patterns is often associated with a poor outcome in patients with various critical conditions 4, 5.

Here, we report a pediatric case of ANE, in which evoked potential studies performed during the acute stage of the disease were useful for predicting functional recovery of the brain.

## Case Report

A 7‐year‐old Japanese boy was transferred to Kitasato University Hospital in December 2013 with new onset of status epilepticus. Two days before transfer, high fever developed. Next day he became lethargic, and he was admitted to another hospital. Rapid diagnostic tests for influenza confirmed the diagnosis of influenza B infection, and he was given intravenous peramivir hydrate. However, the following morning he developed convulsive status epilepticus beginning on the right side of the body and secondarily generalized convulsive seizures; he was then transferred to our hospital.

On arrival (day 1), the patient was in coma; Glasgow Coma Scale score was E1V2M4. The temperature was 40.6°C, the blood pressure 68/30 mmHg, and the pulse 198 beats per minute, and the oxygen saturation was 70% while he was breathing ambient air. His trachea was intubated, and he was admitted to the pediatric intensive care unit with mechanical ventilatory support. Blood‐test results on admission showed metabolic acidosis, leukocytosis, disseminated intravascular coagulation, elevated serum creatine kinase (CK) level (373 U/L), mild hypoglycemia (45 mg/dL), and hepatic and renal dysfunction (AST 227 U/L, ALT 33 U/L, blood urea nitrogen 30.3 mg/dL, creatinine 1.30 mg/dL). The levels of serum CK, AST, and ALT were further elevated on day 4 (AST 8069 U/L, ALT 4140 U/L, and CK 10974 U/L) but subsequently resolved. Cerebrospinal fluid examination revealed seven white blood cells/*μ*L, with mildly elevated protein level (51 mg/dL) and normal glucose level. A brain CT showed symmetrical low‐density areas in the thalamus.

**Figure 1:**
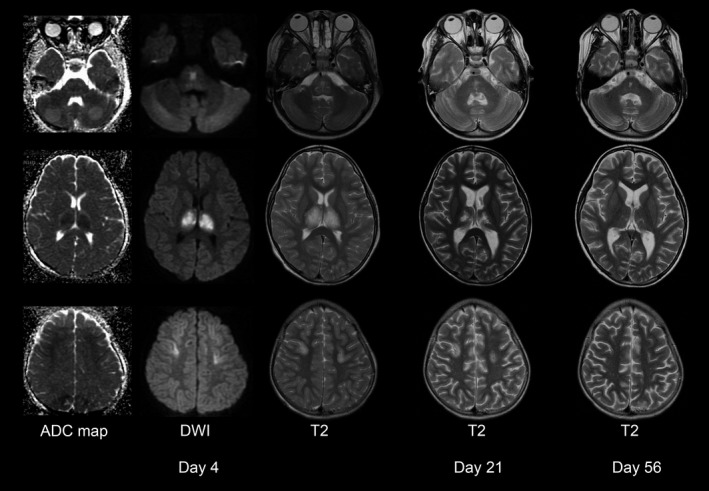
Chronological brain MRI changes. Brain MRIs obtained on day 4 show symmetrical DWI/T2 hyperintensities in the thalamus, frontal cortex, and pons. These brain lesions gradually resolved on day 21 and 56. Note reduction in ADC value in the bilateral thalamic lesions but not apparent changes in other small lesions on ADC map. ADC, apparent diffusion coefficients; DWI, diffusion‐weighted imaging.

**Figure 2:**
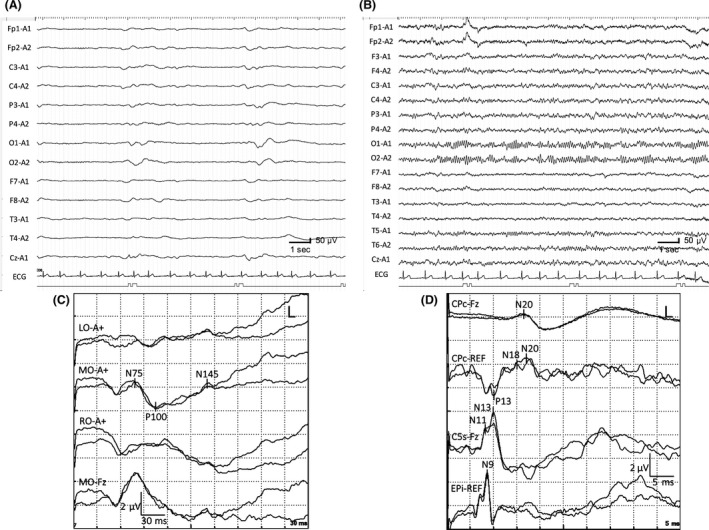
Electrophysiological studies. Panel A, obtained on day 8 after discontinuation of intravenous infusion of midazolam and fentanyl, shows a low‐voltage burst‐suppression pattern. Panel B, obtained after the recovery of symptoms (28 months after the onset of symptoms), shows normal background activity. Panel C, obtained on day 17, shows normal latency and amplitude. Panel D, obtained on day 18, shows normal latency and central conduction time (N13–N20). (A, B) EEG; (C) Flash VEP; (D) SSEP with median nerve stimulation.

The patient was diagnosed with ANE, and he was treated with therapeutic hypothermia (34°C, 48 h), plasma exchanges, intravenous high‐dose methylprednisolone (30 mg/kg/day, 3 days), and intravenous immunoglobulin (1 g/kg/day, 1 day), with continuous infusion of midazolam (0.2 mg/kg/h, 6 days) and fentanyl (2 *μ*g/kg/h, 6 days) from day 1. A brain MRI obtained on day 4 showed symmetrical increased diffusion‐weighted imaging/T2 signals in the thalamus, medial frontal cortex, and pons (Fig. 1). Despite discontinuation of therapeutic hypothermia, the patient remained in coma. An electroencephalogram (EEG) showed irregular delta activity on day 4 while he was being sedated with intravenous midazolam, but low‐voltage burst‐suppression pattern on day 8 despite at least 48 h after discontinuation of sedative drug (Fig. 2A).

In order to evaluate the brain function, we performed a series of evoked potential studies with auditory brainstem response (ABR) on day 8, flash visual evoked potentials (VEP) on day 17, and somatosensory evoked potentials (SSEP) on day 18, respectively. These investigations revealed normal‐appearing evoked potentials (Fig. 2C and D) despite persistent coma lasting 26 days. An EEG obtained on day 38 showed occipital predominant slow alpha activity.

Following the treatments, the patient gradually improved with resolution of brain MRI lesions (Fig. 1). He was transferred to a rehabilitation hospital on day 48, and he ultimately returned back to school 4 months after the onset of symptoms. The follow‐up EEG on day 65 or later showed recovery of background activity (Fig. 2B, 28 months after the onset of symptoms). At the last follow‐up, 48 months after the onset of symptoms, he was able to interact at age‐appropriate level, although his grades at school were not age appropriate due to a mild neurological deficit. The Pediatric Cerebral Performance Category (PCPC) Scale, which was developed to measure morbidity after a child's critical illness or injury 6, was scored 1 of 6 (normal level).

## Discussion

This pediatric case showed that burst‐suppression pattern in a comatose case of ANE with bilateral thalamic necrosis may not always imply a poor long‐term outcome when normal‐appearing evoked potentials are recorded on ABR, VEP, or SSEP during the acute stage of the disease. It is suggested that evoked potential studies may be useful for predicting functional recovery of the brain in patients with ANE. A similar association between evoked potentials and functional outcome using somatosensory evoked magnetic fields has been reported in a case of ANE 7. We also previously reported a similar experience in adult cases of anti‐*N*‐methyl‐d‐aspartate receptor (NMDAR) encephalitis; despite prolonged decreased level of consciousness with diffuse delta slowing on EEG 8, SSEP was evoked normally during the acute stage of the disease, and the patient's long‐term functional outcome was good 9.

One might argue that normal‐appearing ABR or VEP may be related to the absence of brain lesions that disrupt the ascending auditory pathway in the brainstem or visual pathway projecting through the optic nerve to the primary visual cortex. However, it is interesting that, despite bilateral severe thalamic involvements, each evoked potential, such as N13, or N20, was well preserved with normal central conduction time (N13–N20) on SSEP when the patient remained in coma despite the lack of the use of sedative drugs and even 16 days after discontinuation of therapeutic hypothermia. In our case, decreased level of consciousness is more likely caused by severe edema in the thalamus through disruption of ascending reticular activating system and impairment of cognition‐related pathways.

Such thalamic lesions are usually expected to cause irreversible neurological deficits; however, subsequent remarkable recovery of the brain function may be due to a compensatory mechanism associated with younger age at onset, or prompt initiation of aggressively combined immunotherapies, which may have minimalized neuronal damage, thus brain MRI lesions became less marked on the follow‐up studies.

In conclusion, evoked potential studies may be useful in pediatric patients with ANE, not only for the evaluation of the brain function during the acute stage, but also for predicting functional recovery of the brain.

## Ethics

This study is a retrospective observational case report without intervention, which does not require an approval from the Institutional Review Board of Kitasato University.

## Authorship

YO, TI: contributed to study concept or design, data acquisition, analysis or interpretation of the data, drafting/revising the manuscript. ToI, YN, TT, SM: involved in data acquisition, analysis or interpretation of the data, drafting/revising the manuscript. SO, YK: performed data acquisition, analysis or interpretation of the data, drafting/revising the manuscript.

## Conflict of Interest

T. Iizuka: is an editorial board member for Current Treatment Options in Neurology, and Rinsho Shinkeigaku, and received a grant from Japan Epilepsy Research Foundation.

The authors have nothing to disclose regarding conflict of interests or commercial relationships including grants, honoraria, speaker's lists, significant ownership, or financial support from pharmaceutical or other companies.
